# An overview of extrahepatic cholangiocarcinoma: from here to where?

**DOI:** 10.3389/fonc.2023.1171098

**Published:** 2023-05-01

**Authors:** Yongheng Yang, Xiaolu Zhang

**Affiliations:** Department of Physiology and Pathophysiology, School of Basic Medical Sciences, Cheeloo College of Medicine, Shandong University, Jinan, Shandong, China

**Keywords:** extrahepatic cholangiocarcinoma, pathogenesis, tumorigenesis, genomics, tumor microenvironment

## Abstract

Extrahepatic cholangiocarcinoma (eCCA) contains perihilar cholangiocarcinoma and distal cholangiocarcinoma both of which can arise at any point of the biliary tree and originate from disparate anatomical sites. Generally, the incidence of eCCA is increasing globally. Though surgical resection is the principal treatment of choice for the early stages of eCCA, optimal survival remains restricted by the high risk of recurrence when most patients are present with unresectable disease or distant metastasis. Furthermore, both intra- and intertumoral heterogeneity make it laborious to determine molecularly targeted therapies. In this review, we mainly focused on current findings in the field of eCCA, mostly including epidemiology, genomic abnormalities, molecular pathogenesis, tumor microenvironment, and other details while a summary of the biological mechanisms driving eCCA may shed light on intricate tumorigenesis and feasible treatment strategies.

## 1 Introduction

Cholangiocarcinoma (CCA) usually refers to a range of invasive adenocarcinomas including intrahepatic cholangiocarcinoma (iCCA), perihilar cholangiocarcinoma (pCCA) and distal cholangiocarcinoma (dCCA) based on dissimilarly anatomical locations while the latter two are also collectively termed as extrahepatic cholangiocarcinoma (eCCA). Anatomically, pCCA and dCCA can be discriminated by whether the tumor originates between the second-order ducts and the insertion of the cystic duct or from epithelium distal to the insertion of the cystic duct whereas dCCA implicates the common bile duct typically (1). Moreover, pCCA and dCCA also diverge in pathogenesis, cells of origin, genome aberrations, molecular profiles, and risk factors. Although distinct from iCCA, eCCA should be cautiously termed to cover pCCA and dCCA due to the ambiguous origins of pCCA (2). Histologically, pCCA and dCCA are mainly common mucin-producing adenocarcinomas or papillary tumors, unlike more heterogeneous iCCA which can be classified into perihilar large duct type and peripheral small duct type with S100P and SPP1 expressed, respectively, in term of the size or level of the bile duct affected by malignant cells (3–5). Interestingly, the perihilar large duct type of iCCA is more similar to pCCA and dCCA whereas those subtypes can derive from columnar mucin-producing cholangiocytes or peribiliary glands (4). In term of patterns of growth, iCCA tends to be mass-forming while its large duct type and eCCA can be periductal infiltrating or intraductal growing. Besides, several precancerous lesions including mucinous, cystic neoplasm, biliary epithelial neoplasia, intraductal tubulopapillary neoplasm and intraductal papillary neoplasm of the bile duct can be related to iCCA large duct type and eCCA, not iCCA small duct type (4). Furthermore, viral and cirrhosis are usually underlying in iCCA whereas cholangitis and liver flukes are more common in eCCA. Regarding frequent mutations, IDH1 mutations and FGFR2 fusions with targeted drugs are more frequent in iCCA but nearly absent in eCCA which may be inclined to ERBB alterations (4). eCCA is a rare cancer, but its incidence and mortality have been increasing which menace human health severely (6). Regarding the treatment of eCCA, surgical resection with negative margins is the curative and available treatment strategy for patients present with the early-stage or resectable disease when recurrence is still prevalent (7). Moreover, multidisciplinary treatment of advanced eCCA is also crucial. For instance, adjuvant therapy with S-1 encompassing a mixture of tegafu, gimeracil, and oteracil potassium could improve survival among patients with CCA resected according to a phase 3 randomized clinical trial (8). However, effective molecularly targeted therapy for eCCA is still an urgent enigma to be unveiled.

Here, we summarize current advances in the oncogenic mechanisms and treatment strategies of eCCA, mainly concerning epidemiology, genomic abnormalities, molecular pathogenesis, tumor microenvironment, and other pertinent details to provide a comprehensive panorama of eCCA and highlight the importance of personalized and multidisciplinary considerations.

## 2 Epidemiology and risk factors, past and current

The global Incidence of eCCA increased worldwide during the period 1993-2012 spanning two decades according to the CI5plus database for 33 inclusive countries (9). More accurately, the age-standardized incidence for eCCA indeed increased with geographical variation and most evidently in Thailand and Colombia in the 20 years examined. Mortality rates for eCCA have also increased, but more slowly than iCCA in Western countries (6). Summarizing gallbladder carcinoma and other biliary carcinomas including eCCA, an estimated 12,130 new cases, and 4,400 deaths were reported in the United States, in 2022 with a minute difference by gender (10). However, it was also reported that the age-standardized incidence of eCCA has been descending over the past few decades (11, 12). Of note, these trends need conservative assessment given that International Classification of Diseases (ICD) codes for cholangiocarcinoma have been updated several times. Separate codes for iCCA, pCCA, and dCCA were not available until the new ICD-11 classification came into effect which may influence epidemiological estimation (13). Thus, epidemiological trends reported for eCCA need to be evaluated meticulously whereas data is more reliable when ample and new. In addition, pCCA and dCCA have different prognoses and distinctive epidemiological trends. Surveillance, Epidemiology, and End Results (SEER) database have shown better survival in dCCA when compared with pCCA from 2000 to 2018 (14). Regarding dCCA, a recent Swedish cohort study disclosed that incidence rates elevated principally among those patients aged more than 55 during the consecutive calendar periods. Contrastively, the increase in both intrahepatic and perihilar cholangiocarcinoma was more evident in younger adults (15).

In general, several common risk factors including obesity, alcohol consumption, and cigarette smoking could be linked to eCCA (16). Furthermore, metabolic diseases, such as type 2 diabetes, nonalcoholic fatty liver disease, and hypertension are also risk factors for eCCA which are also shared by iCCA (17, 18). Remarkably, dose-dependent alcohol consumption increased the risk of CCA for patients with prediabetes and diabetes, but not normoglycemic, which indicated a synergistic effect, and alcohol abstinence might humiliate the risk of CCA for those patients (19). A large pan-European cohort showed that pCCA was featured with primary sclerosing cholangitis (PSC) and dCCA with choledocholithiasis (20). Though viral infections including hepatitis B virus and hepatitis C virus have been associated with incremental CCA risk previously, they seem to influence iCCA mainly, not eCCA in Europe while a similar situation could be adequate for primary biliary cholangitis (16, 20). Several studies also evaluated the role of drugs such as statins and aspirin in the prevention of eCCA. Statin usage has been noticed to be associated with a reduced risk for eCCA whose users with dCCA had better overall survival than statin-free patients (HR=0.53) (21). Notably, multiple cohorts have revealed that aspirin was associated with a decreased risk of CCA (22, 23). Even so, low-dose aspirin was not associated with eCCA risk significantly but non-steroidal anti-inflammatory drugs with aspirin excluded could increase the risk of eCCA (HR=1.32) as reported by Marcano-Bonilla L et al. (24). Besides, proton pump inhibitors with extended duration may also increase eCCA risk (25). Those evidence indicated that drug usage should be cautious for patients with eCCA.

## 3 Clinical symptoms and diagnosis, early to arise

eCCA can be asymptomatic or non-specific during early stages which makes it tough to diagnose early. The most common symptom of eCCA is obstructive jaundice whereas it is less frequent in iCCA (26). Besides, some constitutional symptoms such as fatigue, anorexia, weight loss, and abdominal pain could be noticed in patients with either benign or malignant diseases (27). Generally, diagnosis of eCCA can benefit from imaging, endoscopy and histology. Imaging techniques including CT and MRI are important for diagnosis and staging of CCA. Owing to direct compression, dCCA shows abrupt biliary tree cutoff from CT scanning while pCCA can be obvious only when dilated segmental bile ducts emerge (28). MRI can delineate the biliary tree with its lesions in detail and allow accurate ducts depicted by magnetic resonance cholangiopancreatography (MRCP) which is critical for the diagnosis, staging, and treatment planning of pCCA (29). MRI illustrates CCA as hypointense lesions and heterogeneously hyperintense lesions on T1-weighted images and T2-weighted images, respectively (30). Remarkably, Endoscopic retrograde cholangiopancreatography (ERCP) is a robust mode for the biliary tree assessment and acquirement of brush cytology and biopsies with high specificity but low sensitivity (31). In addition to the primary modalities including MRCP and ERCP, endoscopic ultrasound (EUS) can be complemental and helpful for the evaluation of biliary strictures and assessment of eCCA or regional lymph nodes (32). It also allows tissue acquisition *via* needle aspiration and may detect small bile duct masses (33). Furthermore, cholangioscopy covering bile duct mucosa and targeted biopsies could enhance the diagnostic accuracy of malignant biliary strictures (34). Recently, Ishii T et al. reported that cholangioscopy enhanced by image systems is very useful for diagnosing eCCA (35). Histologically, eCCA can be flat, nodular sclerosing, or intraductal papillary type whose growth patterns are periductal infiltrating or intraductal growing. eCCA derives from mucous cells and/or columnar cholangiocytes which also concern precancerous lesions including intraductal epithelial neoplasia. Several tissue markers such as MUC5AC, MUC6, S100P, and BAP1 contribute to differentiating eCCA from diverse CCA types (4). In total, early diagnosis is still challenging for eCCA and a combination of clinical, imaging, endoscopy and histologic data is usually necessary.

## 4 Surgical resection and adjuvant therapy, two rocks and one bird

Surgical resection maintains a momentous tactic for pCCA and dCCA therapy while adequate assessment and preoperative consideration are necessary to be priorly executed which restricts candidates for curative-intent surgical resection therapy (36, 37).

Generally speaking, pancreaticoduodenectomy and lymphadenectomy are involved in surgery for dCCA (**Table 1**). Achieving a margin negative (R0) resection is crucial for dCCA and pCCA management while negative margin assessment and complete resection may benefit from the intraoperative frozen section (43). Curative and eligible surgical resections for eCCA patients depend on multiple clinical conditions. A study based on a cohort in the Netherlands determined an overall survival predictive model for patients after pancreatoduodenectomy for dCCA. Five independent prognostic factors covering age at diagnosis, pT category, pN category, resection margin status, and tumor differentiation were included in the model which was also robust for inferring prognosis (44). Furthermore, both tumor budding and tumor invasive thickness were associated with adverse postoperative prognosis in eCCA (45, 46). Interestingly, nerve fiber density invaded by tumors could be related to unfavorable outcomes of pCCA patients undergoing curative-intent surgery (47). Regarding preoperative evaluation, preoperative biliary drainage is still debated but needed when obstructive symptoms are present for eCCA patients whereas endoscopic biliary drainage seems to be more suitable for dCCA than percutaneous transhepatic biliary drainage which had lower rates of complications for pCCA (48–50). Moreover, laboratory assessment on peripheral blood revealed that neutrophil count, fibrinogen-to-lymphocyte ratio (FLR), and FLR-neutrophil score could predict the prognosis of patients with resected eCCA (51).

**Table 1:** Effective therapeutical procedures for extrahepatic cholangiocarcinoma.

Procedures	Details	Reference
*Surgical resection*	Pancreaticoduodenectomy, lymphadenectomy	(36, 37)
*Adjuvant therapy*	Radiotherapy, chemotherapy	(38, 39)
*Endoscopy*	Radiofrequency ablation, stent	(40)
*Targeted therapies*	EGFR/ERBB2 inhibitors	(41)
*Immunotherapy*	Anti-PD1 and/or anti-PD-L1	(42)

Historically, adjuvant therapy after curative resection of biliary tract cancer is commendatory whose decisions need to be based on adequate and robust data from clinical trials. Previously, no difference was settled between the gemcitabine adjuvant chemotherapy group and the control group in eCCA patients who underwent curative resection from a randomized phase 3 trial (52). Recently, another randomized phase 3 trial confirmed adjuvant therapy with S-1 (a mixture of tegafu, gimeracil, and oteracil) could improve survival among patients with resected eCCA, iCCA, gallbladder carcinoma (GBC), and ampullary carcinoma involved versus surgery alone (8). A prospective study (SWOG 0809) focusing on adjuvant chemotherapy (gemcitabine and capecitabine) followed by chemoradiation in patients with eCCA and GBC showed that adjuvant therapy could benefit patients with lymph node-positive (53). Similarly, adjuvant therapy could improve the long-term survival of patients with perineural invasion and lymph node metastasis after curative-intent resection for dCCA (38). Although phase 3 studies evaluating adjuvant radiotherapy are lacking, there are shreds of evidence that adjuvant radiotherapy should be considered for patients after resection of dCCA (39). To sum up, the role of neoadjuvant and adjuvant therapies for eCCA should be optimized with more comprehensive investigations (**Table 2**).

**Table 2:** Robust clinical trials of extrahepatic cholangiocarcinoma.

Approach	Sample size	Agents	Clinical trial ID	Reference
Adjuvant chemotherapy	225	Gemcitabine	UMIN 000000820	(52)
Adjuvant chemotherapy	69	Gemcitabine and Capecitabine	SWOG 0809	(53)
Endoscopic radiofrequency ablation	65	NA	NCT02592538	(54)
Endoscopic radiofrequency ablation	174	NA	NCT01844245	(55)
Endoscopic radiofrequency ablation	75	S-1	NCT02592538	(56)
Chemotherapy plus targeted therapy	133	Gemcitabine and Oxaliplatin plus Erlotinib	NCT01149122	(57)
Chemotherapy plus targeted therapy	122	Gemcitabine and Oxaliplatin plus Cetuximab	NCT01267344	(58)
Chemotherapy plus targeted therapy	90	Cisplatin and Gemcitabine plus Panitumumab	NCT01320254	(59)
Chemotherapy plus targeted therapy	85	Gemcitabine and Oxaliplatin plus Panitumumab	NCT01389414	(60)
Immunotherapy	104	Pembrolizumab	NCT02628067	(61)
Immunotherapy	54	Nivolumab	NCT02829918	(62)
Immunotherapy	77	Atezolizumab plus Cobimetinib	NCT03201458	(63)

For patients with unresectable disease, the FDA has approved pembrolizumab for patients with unresectable or metastatic microsatellite instability-high or mismatch repair deficient solid tumors (including CCA) (37). However, as shown in results from the KEYNOTE-158 and KEYNOTE-028 studies, pembrolizumab treatment achieved a low objective response rate of 6–13% and an inferior survival of less than 2 months in patients (61). Remarkably, liver transplantation (LT) is a therapeutic option in patients with unresectable malignant tumors including CCA (37). However, early experience showed high recurrence rates with transplant (64). Despite poor outcomes after LT for CCA, recent studies have fluctuated this premise since neoadjuvant therapy including chemotherapy and/or radiotherapy followed by liver transplantation offers a potentially curative strategy for patients with unresectable disease (65). For instance, a recent meta-analysis indicated that LT with neoadjuvant chemoradiation completed achieved higher overall survival rates than LT alone in patients with unresectable pCCA (82.8%, 65.5%, and 65.7% at 1 year, 3 years, and 5 years, respectively, vs. 71.2%, 48%, and 31.6%, respectively; p < 0.001) (66). It further supports the curative possibility of neoadjuvant chemoradiation therapy followed by liver transplantation for unresectable CCA patients.

Regarding the management of complications including obstructive jaundice and biliary infection for unresectable eCCA, endoscopic biliary stent placement is effective partially, but limited in improving the overall survival of patients (67). Endoscopic radiofrequency ablation (RFA) has been an accessible technique for alleviating malignant biliary stenosis since first reported (40), although it may be inclined to treat patients without distant metastasis (68, 69) (**Table 1**). Several randomized controlled trials showed that additional endoscopic RFA could improve the overall survival of patients with unresectable eCCA than those with stent placement alone (54, 55). Furthermore, endoscopic RFA combined with S-1 administered orally for unresectable eCCA patients achieved significantly longer survival (16 months vs. 11 months, p<0.01) and stent patency time (6.6 months vs. 5.6 months, p=0.014) than RFA sole (56). Evidence from retrospective studies also indicated that patients with locally advanced eCCA could benefit from the combination of endobiliary RFA and gemcitabine plus cisplatin treatments (70, 71).

## 5 Tumor microenvironment of eCCA, no cell is alone

The tumor microenvironment (TME) is composed of diverse cellular types and extracellular components, supporting and maintaining tumor progression while deciphering the complexity of TME is more feasible in the single-cell era (72).

**Figure 1:**
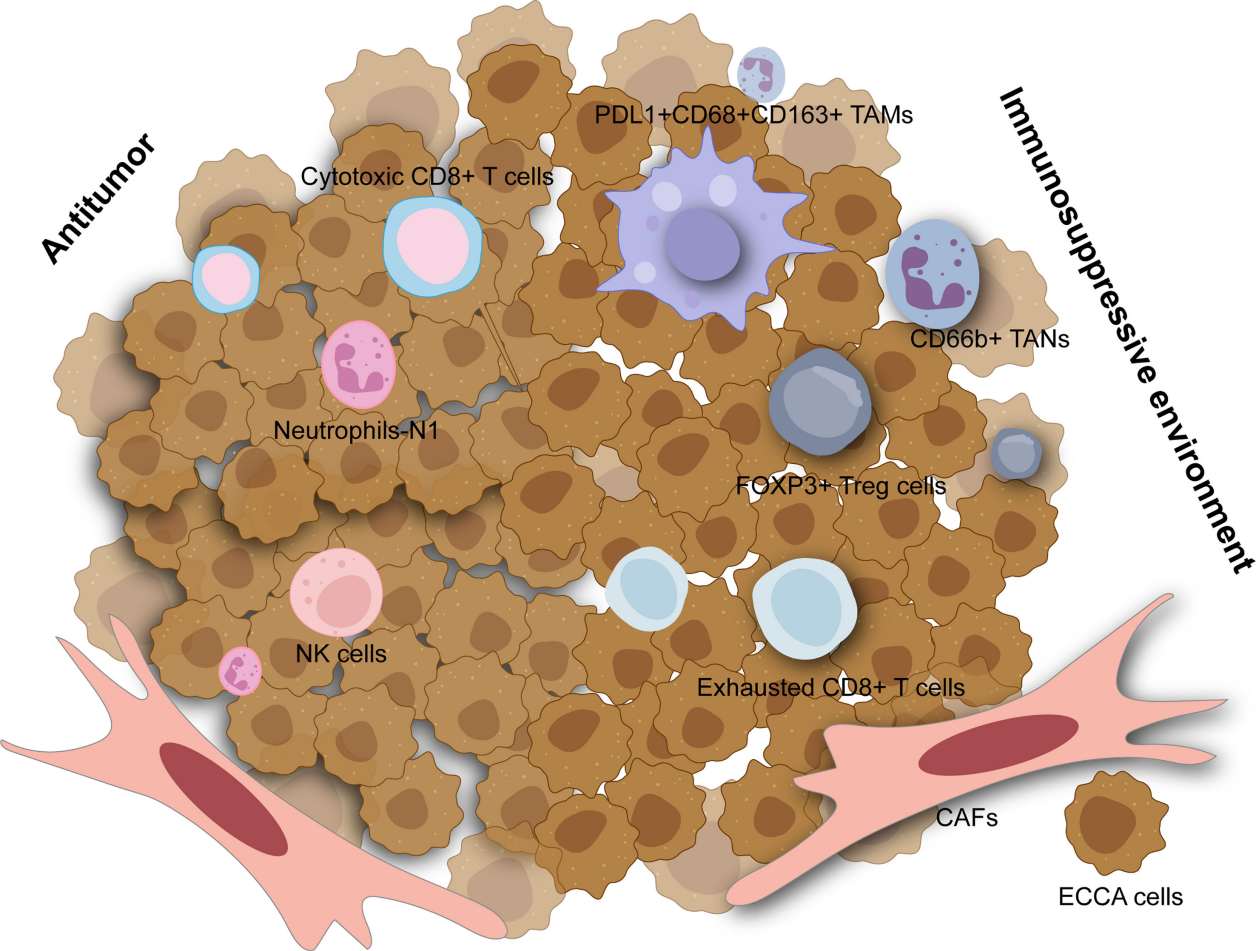
The tumor microenvironment in extrahepatic cholangiocarcinoma. The immunosuppressive environment is mainly composed of tumor-associated macrophage (TAMs), tumor-associated neutrophils (TANs), regulatory T cells (Tregs), exhausted CD8+ T cells and cancer-associated fibroblasts (CAFs) which are all associated with worse outcomes. Cytotoxic CD8+ T cells, neutrophils with N1 phenotype, and natural killer (NK) cells can have antitumor effects in extrahepatic cholangiocarcinoma.

Among innate immune cells, activated M2 macrophages induce tumor progression with anti-inflammatory and immunosuppressive effects which could stimulate the canonical Wnt/b-catenin pathway driving CCA growth (73). A high density of tumor-associated macrophages was associated with incremental recurrence of pCCA in a retrospective study (74). Furthermore, elevated PD-L1+ M2 tumor-associated macrophages (CD45+ CSF1R+ CD68+ CD163+) also correlated with inferior outcomes in dCCA and higher expression of IL6, IL10, and ARG1, contributing to effector T cell suppression (**Figure 1**) (75). Though natural killer (NK) cells may comprise a considerable proportion across immune ingredients of eCCA and seem to be lower in tumors compared to para-tumor tissues and peripheral blood (76), they were insufficiently studied in eCCA. A previous study reported that a mouse xenograft model induced by HuCCT-1 cells, an iCCA cell line, and then infused with *ex vivo* expanded human NK cells showed significant tumor inhibition (77). Tumor-associated neutrophils (TANs, CD66b+) could predict poor prognosis in eCCA patients (78). Similarly, the systemic immune-inflammation index calculated by neutrophil, platelet, and lymphocyte counts from serum was an independent prognostic factor for patients under resection of eCCA (79). Interestingly, neutrophils recruited by tumor-cell-derived microvesicles loading methotrexate and subsequent macrophage repolarization could alleviate biliary obstructions of patients with eCCA and execute tumor cells with reactive oxygen species and nitric oxide levels elevated, displaying an antitumor N1 phenotype (80). However, neutrophils heterogeneity in eCCA is still poorly understood. Remarkably, immunosuppressive functions including recruiting macrophages and suppressing T cell cytotoxicity of TANs have been elucidated adequately in liver cancer at the single cell resolution recently (81).

Regarding the adaptive immune system, tumor-infiltrating lymphocytes (TILs) mainly include CD4+ T lymphocytes and CD8+ T lymphocytes which consist of diverse subsets in eCCA (82). FOXP3+ regulatory T cells (Tregs) characterized by TGF-β and IL-10 secretion are noticed to infiltrate into the tumors with an immunosuppressive profile. Several studies have elevated Tregs in eCCA based on immunohistochemical results for FOXP3 while increased Tregs are significantly associated with worse OS in patients with p/dCCA (78, 83, 84). Experiments *in vitro* showed that FOXP3+ Tregs could be recruited by FOXM1 which bound to the FOXP3 promoter region and thus promoted its transcription in pCCA cell lines (85). Similarly, single-cell RNA sequencing on tissues derived from patients with dCCA also revealed that tumor infiltrating Tregs were abundant in dCCA tumors with immunosuppressive genes such as TIGIT, CTLA4, and TNFRSF18 highly expressed (**Figure 1**) (86). Furthermore, several genes related to immunotherapy including ACP5, MAGEH1, TNFRSF9, and CCR8 could be specially expressed in tumor infiltrating Tregs in eCCA as shown in the single-cell landscape from another research (76). For CD8+ T cells, some studies concluded that higher numbers of them were associated with better OS for eCCA (78, 82) while heterogeneity of CD8+ T cells may be neglected. As reported recently, cytotoxic CD8+ T cells could function as effectors in dCCA while exhausted CD8+ T cells were also enriched with PDCD1, CTLA4, LAG3, and HAVCR2 expressed (76, 86). Notably, mucosal-associated invariant T cells possessing cytotoxicity and innateness were absent in the pCCA tumor microenvironment (87). Histologically, canonical tertiary lymphoid structures were associated with favorable survival in pCCA (88).

Cancer-associated fibroblasts (CAFs) are a heterogeneous cell population of fibroblasts and myofibroblast-like cells and constitute CCA stroma chiefly with typical phenotypic markers such as α-SMA, PDGFRβ, FAP, and so on (89). In CCA, CAFs likely derive from a variety of cell types including hepatic stellate cells, portal fibroblasts, fibrocytes, or epithelial mesenchymal transition (EMT) (90). CAFs can mediate crosstalk with CCA cells or TME which pave the road for tumorigenesis. Extrahepatic TFK-1 cells co-cultured with CAFs showed incremental activation of STAT3, JNK, ERK, and AKT pathways (91). Admittedly, recent studies focused on CAFs and iCCA more while some evidence was also not special for eCCA (92, 93).

## 6 Genomic landscape of eCCA, common and maverick

**Figure 2:**
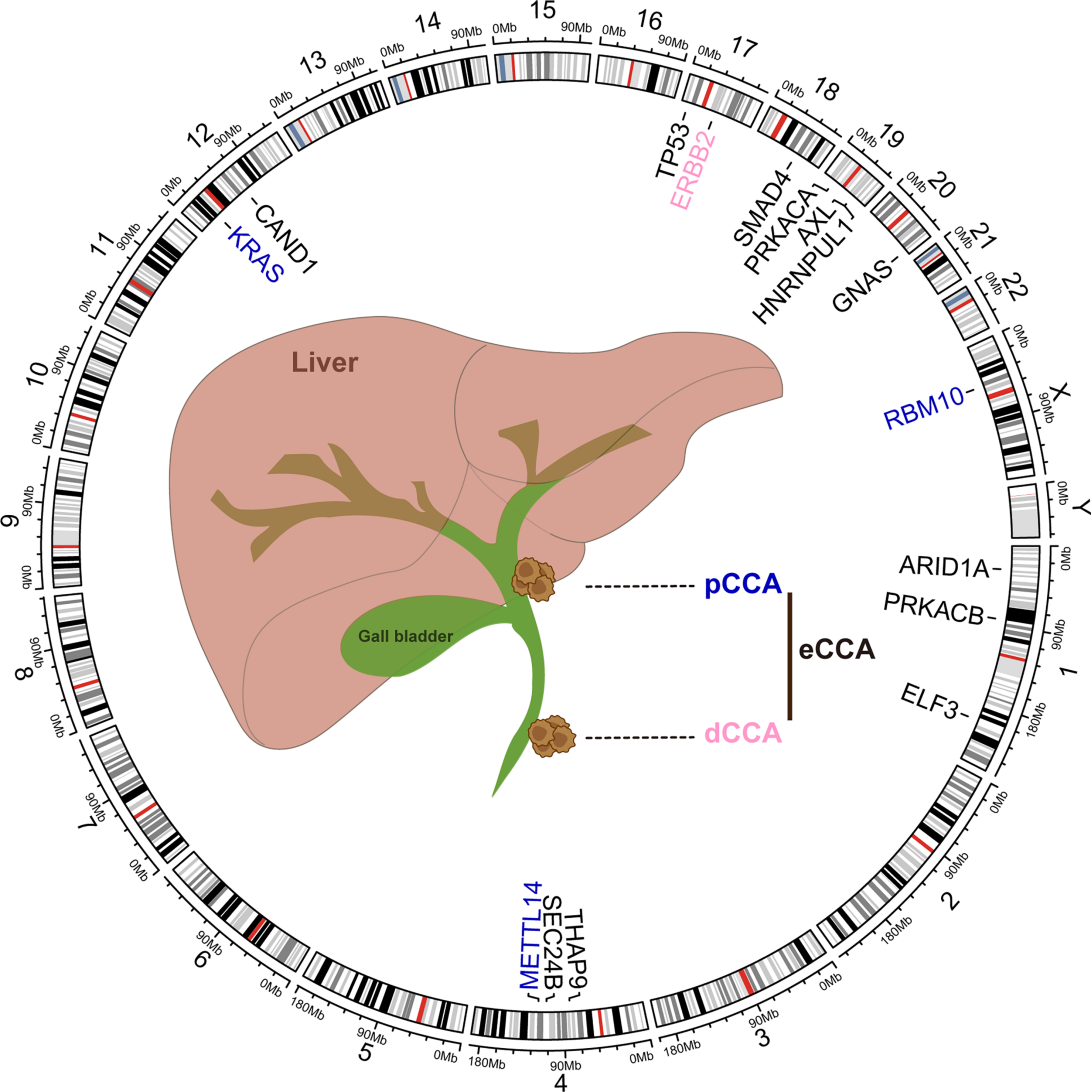
Common genomic alterations in extrahepatic cholangiocarcinoma. The genetic alterations and locations in human chromosomes (hg38) were depicted in the circos plot executed by R language package criclize.

Molecular heterogeneity across eCCA has been unveiled at the genomic level whereas pCCA and dCCA do bear dissimilar genomic alterations (94). DNA mismatch repair (MMR) deficiency could be found in about 5% of pCCA and dCCA, lower than iCCA as reported previously (95). Conventional mutations in TP53, KRAS, ARID1A, SMAD4, and GNAS were commonly shared in eCCA whereas CCA subtypes do carry diverse genomic profiles (96, 97). PRKACA and PRKACB fusions and ELF3 mutations could be inclined to occur in pCCA/dCCA (98). According to Simbolo M et al, KRAS mutations may be more prevalent in dCCA when compared to pCCA (99). Paradoxically, KRAS mutations were more common in pCCA than dCCA in another cohort (94). Furthermore, ERBB2 amplifications could occur more frequently in eCCA (100). ERBB2 mutations or amplifications were also linked to a proliferation class of eCCA where patients with dCCA predominate (96). Several driver genes involved in post-transcriptional modification such as RBM10 and METTL14 mutation were more enriched in pCCA than iCCA. Conversely, both tumor mutation burden and copy number alteration burden of pCCA were lower than iCCA (101). Intriguingly, aristolochic acid exposure which could induce TP53 mutation in iCCA was superior to eCCA in a Chinese cohort where high mutational frequencies of THAP9, SEC24B, and CAND1 were noticed in eCCA (102). Actually, canonical FGFR2 fusion events were nearly absent in eCCA whereas AXL-HNRNPUL1 gene fusions could be detected in a few cases with eCCA (98, 100). Of note, cell-free DNA (cfDNA) analysis excels at shedding light on tumor heterogeneity and provides an unbiased genomic profiling for patients. cfDNA analysis on advanced cholangiocarcinoma (both iCCA and eCAA, subtype was not specified) revealed that three targetable alterations including FGFR2 fusion, IDH1 mutations, and BRAF V600E were clonal in the generality of the cohort. Besides, discordance and concordance between cfDNA and tissue for mutation detection could be noticed in the former one and the latter two, respectively (103). The high heterogeneity of eCCA can be likely attributed to genomics aberrations, highlighting the demand for characterizing the molecular basis of sensitivity and resistance to available treatments (**Figure 2**).

## 7 Pathogenesis of eCCA, classical but complex

**Figure 3:**
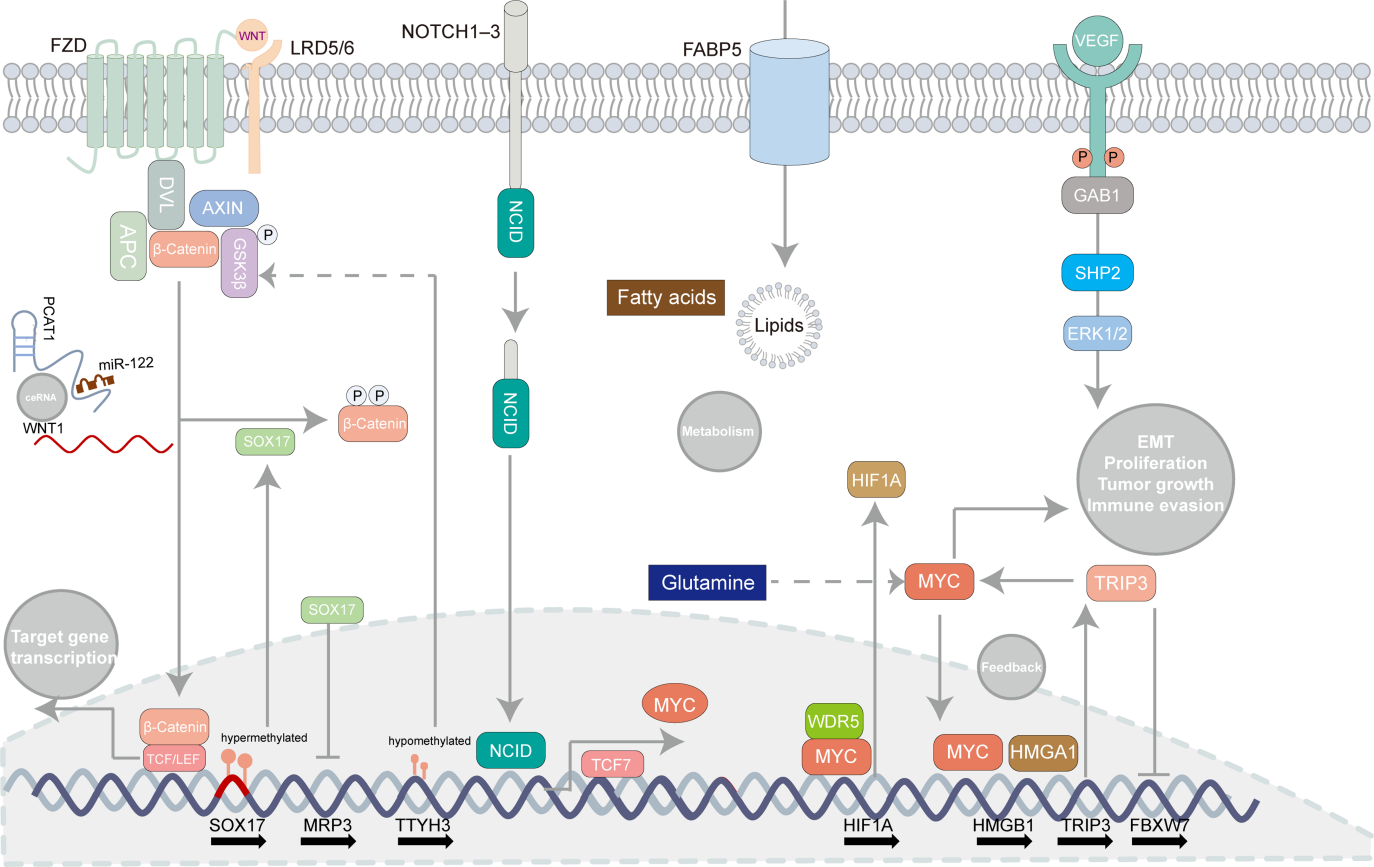
Oncogenic pathways involved in extrahepatic cholangiocarcinoma. Some canonical pathways can be related to tumorigenesis in extrahepatic cholangiocarcinoma such as the Notch signaling pathway, the WNT/β-catenin pathway, the MYC-oncogene pathways, lipid metabolism, and angiogenesis. Several regulatory mechanisms concerning those pathways are recapitulated in the illustrator.

To elaborate pathogenesis of eCCA is insurmountable while it is challenging to catch the “Achilles’ Heel” of eCCA which can be related to signaling pathways to some extent. According to bulk transcriptomic profiles, ‘metabolic’, ‘proliferation’, ‘mesenchymal’, and ‘immune’ subtypes of eCCA were previously identified with disparate oncogenic pathways activated respectively (96). Indeed, several developmental pathways can be linked to eCCA (**Figure 3**). The Notch signaling pathway counts on ligands attaching to Notch receptors and subsequent release of Notch intracellular domain 1 (NICD1) which is then shifted to the nucleus where target genes regulating cell proliferation, migration, and invasion are activated (2). Although recent research mainly focused on the mechanism of the Notch pathway and iCCA (104), the Notch receptors were indeed overexpressed in pCCA and dCCA (105). The Wnt/β-catenin pathway is commonly activated in CCA and partially mutated in dCCA (73, 106). The Wnt/β-catenin pathway could be inhibited through ClC-5 inhibition in eCCA cells (107). TTYH3 could facilitate cell proliferation, migration, and invasion *via* the Wnt/β-catenin pathway in the QBC939 cell line (108). lncRNA PCAT1 was also involved in the positive regulation of pCCA and dCCA progression through miR-122 (109). Remarkably, SOX17 which is the WNT/β-catenin pathway promoter inhibitor was hypermethylated and thus repressed in patients with CCA (110). Apart from its seeming tumor suppression effect, SOX17 could sensitize tumors to chemotherapy with MRP3 suppressed in EGI-1 and TFK-1 cell lines (111). Alteration of classic oncogenic pathways is also involved in the pathogenesis of eCCA with genomic instability (96). For instance, transcription factor HOXA5 could augment MXD1 expression by binding to its promoter region directly which then activated the p53 signaling pathway, thus inhibiting eCCA cell proliferation (112). Notably, the MYC-oncogene pathways can drive tumorigenesis and be related to immune evasion in cancer (**Figure 3**) (113). HMGA1 inducing TRIP13 expression which suppressed FBXW7 transcription could stabilize c-Myc which expedited their transcription in a positive feedback, thus promoting EMT and stemness of pCCA (114). TCF7 inducing c-Myc transcription could impel pCCA progression (115). Besides, WDR5 could boost HIF-1α accumulation and then drive EMT and metastasis of eCCA in a Myc-dependent way (116). Interestingly, the depletion of glutamine could offset hypoxia-induced chemoresistance in eCCA cells with c-Myc restraint (117). Regarding metabolism pathways, lipid metabolism, and fatty acid oxidation were strikingly enhanced in the EGI1 cell line with intracellular lipids accumulation and increased cell stemness (118). Compared with iCCA, FABP5 functioning as a fatty acid transport protein is highly expressed and associated with poor survival in eCCA (119). Moreover, JNK/c-Jun pathways could also be associated with both iCCA and eCCA (120, 121). Proinflammatory cytokines, such as IL-6, IL-8. can be involved in augmenting tumorigenesis of eCCA. IL-6 in serum was a prognostic factor in eCCA patients (122). Likewise, the Genetic variant of CXCR1 (also termed IL-8RA) could predict inferior outcomes for pCCA patients (123). Angiogenesis is also essential for eCCA. High levels of VEGF have been noticed in eCCA cell lines and tissues previously (124). Recently, Li T et al. reported that VEGF was regulated by Gab1 *via* SHP2/ERK1/2 which could be inhibited by apatinibin in pCCA cells (125).

Cancer stem cells (CSCs) are a characteristic subpopulation of tumor cells and harbor the ability to maintain renewal which can be involved in recurrence, metastasis, and drug resistance (126). As shown in accumulative shreds of evidence, CSCs are interrelated with EMT intimately (4). Not only does TGFβ contribute to EMT, but it also facilitates stemness in extrahepatic TFK-1 cells *in vitro* (127). Besides, CSCs from iCCA and eCCA can be identified with ALDH expressed (127). Remarkably, though cell proliferation and invasion were more increased in iCCA than in eCCA cell lines, stem cell surface markers (CD13, CD24, CD44, CD90, and EPCAM) were similarly expressed for both sides (128).

## 8 Biomarkers of eCCA, novel or clinical

**Figure 4:**
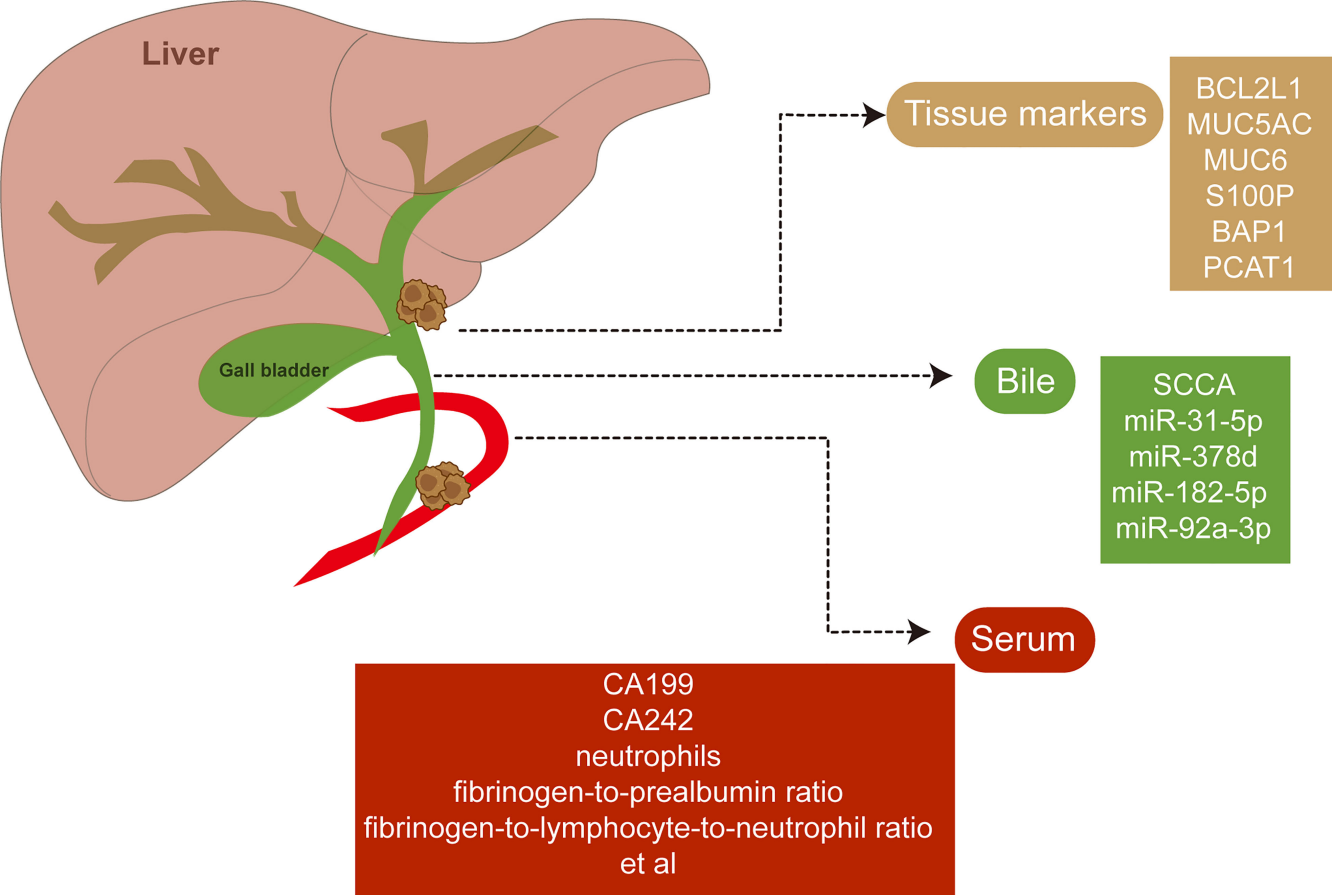
Potential biomarkers of extrahepatic cholangiocarcinoma. Novel biomarkers of extrahepatic cholangiocarcinoma have been discovered with abundant specimens including tissues, bile, and serum available.

Non-invasive and robust biomarkers of eCCA with diagnostic and prognostic significance have been urgent for execution. Novel biomarkers of eCCA have been emerging with advanced test tools and abundant specimens available (**Figure 4**). Squamous cell carcinoma antigen (SCCA) detected in bile samples was found to be increased in patients with eCCA and could be a special biomarker for eCCA (129). Similarly, microRNA (miR-31-5p, miR-378d, miR-182-5p, and miR-92a-3p) derived from bile cytologic samples were also increased in eCCA cases compared with control cases (130). Anti-apoptotic protein Bcl-x_L_ encoded by BCL2L1 was identified as a prognostic marker in cholangiocarcinoma depending on anatomical subtypes when it indicated beneficial prognosis, especially for pCCA (131). The preoperative serum is also an accessible and robust source for biomarkers in biofluids. Preoperative serum carbohydrate antigen 19-9 could be related to regional lymph node metastases and the prognosis of both pCCA and dCCA with a cutoff of 37 U/ml (132–134). A recent study reported that elevated serum CA242 (>20 IU/ml) was associated with vascular invasion and lymph node metastasis of pCCA (135). Furthermore, inflammatory markers including neutrophils, fibrinogen-to-prealbumin ratio, and fibrinogen-to-lymphocyte-to-neutrophil ratio from preoperative peripheral blood were all independent factors for overall survival of eCCA according to the recent multivariate Cox analyses (51).

Moreover, long non-coding RNAs (lncRNAs) are characterized as non-coding RNAs whose transcripts are longer than 200 nucleotides lacking the ability to code for proteins but influencing tumorigenesis, which are also implicated in the molecular biomarkers of CCA (136, 137). For instance, lncRNA PCAT1 was remarkably upregulated in both eCCA tissues and cell lines (109). MALAT1 could be involved in the pathogenesis of pCCA and predict poor overall survival (138). Some studies have also evaluated the role of lncRNAs in eCCA cell lines. AFAP1-AS1 was relevant to cell growth and metastasis in TFK-1 cell line (139). LINC00184 increased cell growth in QBC939 cell line (140). However, effective tissue markers related to lncRNA for identify eCCA are yet to be discovered.

## 9 Rare histological subtypes related to eCCA, less is more

Histologically, pCCA and dCCA mainly cover mucinous adenocarcinomas or papillary tumors beyond which several additional histological subtypes could be also noticed in eCCA, rarely but factually (141). Adenosquamous carcinoma featured with concomitant adenocarcinoma and squamous carcinoma accounts for 2% of eCCA as previously reported (142). Though it occurs predominantly among the rare subtypes of eCCA, adenosquamous carcinoma can carry different molecular profiles (143). A recent case report showed that an adenosquamous carcinoma patient with distant lymph node metastases carried Her-2 amplification and preserved a stable state after receiving several lines of trastuzumab treatment combined with chemotherapy and radiotherapy (144). Besides, another rare type related to eCCA is signet ring cell carcinoma. Signet ring cell carcinoma is characterized by abundant mucus in the cytoplasm extruding nucleus from center to margin of cell. Generally, a few cases with signet ring cell carcinoma of eCCA were reported up to now (145). Previous studies also described two separable types containing intestinal type and pancreatobiliary type with CK7 negative plus CK20/MUC2 positive and CK7 positive plus CK20/MUC2 negative, respectively (146, 147). That signet ring cell carcinoma of eCCA with distant lymph node metastasis has also been noticed recently (145). Distant metastases always lead to a poor prognosis in eCCA patients. A SEER-based study reported that the liver and distant lymph were the most common sites for metastases and multiple sites (at least two) occurred in some cases (148). Particularly, patients with unresectable advanced eCCA and liver metastases may benefit from chemotherapy combining gemcitabine and cisplatin or pembrolizumab and nab-paclitaxel (149, 150). About the gastrointestinal tracts, several studies also reported colonic metastasis of eCCA (151, 152). Rarely, distal skeletal muscle metastasis could appear in a few eCCA patients as reported (153). Those evidence suggested that adequate follow-up periods should be considered for eCCA since sporadic metastasis could occur.

## 10 Targeted therapies and immunotherapy

Molecularly in-depth understanding of CCA contributes to confirming achievable drug targets. Although IDH1 mutations and FGFR2 fusions do provide new treatment tactics, they are more frequent in iCCA and nearly absent in eCCA (100, 154, 155). Moreover, several randomized controlled trials concerning the epidermal growth factor receptor (EGFR) inhibitors (erlotinib, cetuximab, lapatinib, or panitumumab) did not achieve effective outcomes in advanced CCA (57–60, 156) (**Table 2**). A previous meta-analysis has also shown that first-line chemotherapy with the addition of anti-EGFR monoclonal antibodies does not improve the overall and progression-free survival of patients with advanced CCA (157). Alternatively, targeting abnormal ERBB2 which is more common in eCCA may be a favorable approach. A case report suggested a combination of Trastuzumab and pertuzumab was curative for the patient with ERBB2-amplified eCCA (41). Immune checkpoint blockade can reinforce antitumor immunity by hindering intrinsic suppressors (e.g. CTLA4, PD1, or PDL1) from the immunosuppressive microenvironment where the tumor locates while several checkpoint inhibitors have been approved for clinical application (158). Regarding eCCA, four novel transcriptome-based subtypes have been suggested (96). Tumors in the “immune” class not only overexpressed PD-1/PD-L1 but also had a higher lymphocyte infiltration which implies a better response to immune checkpoint inhibitors. Furthermore, the ratio of PD1 positive to CD8 + TILs could be linked to worse outcomes for eCCA patients (159). A subset of CD8+RORγt+ T cells with PD1 expressed lowly was noticed to be associated with reduced survival in dCCA as reported previously (160). Actually, pembrolizumab seems to be more effective in CCA patients with microsatellite instability (MSI) or mismatch repair deficiency (dMMR) whose incidence is low in CCA while it is also reported that the number of ECC patients with PDL1 positive could be small (161, 162). Indeed, the TOPAZ-1 trial has improved our understanding of CCA and immunotherapy (42).

Several immunotherapy agents such as Pembrolizumab, Nivolumab and Atezolizumab have shown low response rates in patients with advanced stages of CCA (61–63).

Up to now, more clinical trials are still requisite for eCCA.

## 11 Conclusion

CCA is heterogeneous and comprised of diverse subtypes. Not only do those subtypes arise from different locations, but iCCA and eCCA also carry disparate risk factors, diverse cells of origin, and individual genome aberrations. Sophisticated interactions between eCCA cells or CSCs, and the TME make it laborious to elaborate the biological mechanisms underpinning tumorigenesis where high-resolution single cell multi-omics may shed light on. Now, there is still a lack of therapeutic approaches for eCCA since not all patients with eCCA can benefit from accessible treatments including surgery, adjuvant therapy, targeted therapies, and immunotherapy, emphasizing the importance of personalized and multidisciplinary considerations. However, improved understanding of the specific TME and pathogenesis in eCCA, along with accumulating data from single cell resolution will indisputably bring more efficient therapeutic options for patients in the future. Furthermore, considering that several benign diseases are risk factors of eCCA, it is also crucial for patients with eCCA to prevent early, diagnose accurately, and treat timely. That is, better to batten down the hatches before the storm comes.

## Author contributions

YY: Visualization, data curation, writing - original draft. XZ: Project administration, supervision, writing - review & editing. Both authors contributed to the article and approved the submitted version.
